# A Importância da Manobra de Valsalva Eficaz durante a Ecocardiografia na Miocardiopatia Hipertrófica

**DOI:** 10.36660/abc.20230871

**Published:** 2024-07-01

**Authors:** Viviane Tiemi Hotta, Minna Moreira Dias Romano, Silvio Henrique Barberato, Marcelo Luiz Campos Vieira, Fábio Fernandes, Marcus Vinicius Simões

**Affiliations:** 1 Universidade de São Paulo Faculdade de Medicina Instituto do Coração do Hospital das Clínicas São Paulo SP Brasil Instituto do Coração do Hospital das Clínicas da Faculdade de Medicina da Universidade de São Paulo, São Paulo, SP – Brasil; 2 Universidade de São Paulo Faculdade de Medicina de Ribeirão Preto Ribeirão Preto SP Brasil Universidade de São Paulo Faculdade de Medicina de Ribeirão Preto, Ribeirão Preto, SP – Brasil; 3 CardioEco Centro de Diagnóstico Cardiovascular Curitiba PR Brasil CardioEco Centro de Diagnóstico Cardiovascular, Curitiba, PR – Brasil; 4 Quanta Diagnóstico – Ecocardiografia Curitiba PR Brasil Quanta Diagnóstico – Ecocardiografia, Curitiba, PR – Brasil; 5 Hospital Israelita Albert Einstein – Ecocardiografia Adultos São Paulo SP Brasil Hospital Israelita Albert Einstein – Ecocardiografia Adultos, São Paulo, SP – Brasil

**Keywords:** Cardiomiopatia Hipertrófica, Ecocardiografia, Manobra de Valsalva

A manobra de Valsalva foi descrita pela primeira vez em 1704 como uma expiração forçada contra nariz e glote fechados.^1,2^ Naquela época, nenhuma observação dos efeitos circulatórios foi mencionada. Em 1853, Weber et al. descreveram os efeitos hemodinâmicos e circulatórios relacionados à manobra de Valsalva, e desde então ela tem sido amplamente utilizada na cardiologia.^3^ As aplicações iniciais da manobra de Valsalva focaram na investigação do controle do reflexo autonômico cardíaco.^4^

Atualmente, a manobra de Valsalva é recomendada na ecocardiografia, em cenários específicos, como em pacientes com miocardiopatia hipertrófica (MCH), para avaliação de obstrução dinâmica da via de saída do ventrículo esquerdo (VSVE). Porém, há grande variação na sua execução e falta de padronização de controle de pressão e tempo de execução.^5^

Particularmente, em pacientes com MCH, é crucial avaliar o gradiente da VSVE por meio de testes provocativos em pacientes sem obstrução significativa da VSVE em repouso, uma vez que a grande maioria dos pacientes sintomáticos com MCH apresenta obstrução da VSVE em repouso ou provocada como mecanismo fundamental de dispneia, o que pode ser um alvo terapêutico relevante.

Como a obstrução da VSVE é extremamente influenciada pelas condições de carga ventricular esquerda,^6^ as alterações hemodinâmicas associadas à manobra de Valsalva favorecem seu uso como teste provocativo em pacientes com MCH. A Figura 1 mostra as alterações da pressão arterial e da frequência cardíaca durante as diferentes fases da manobra de Valsalva.

**Figura 1:**
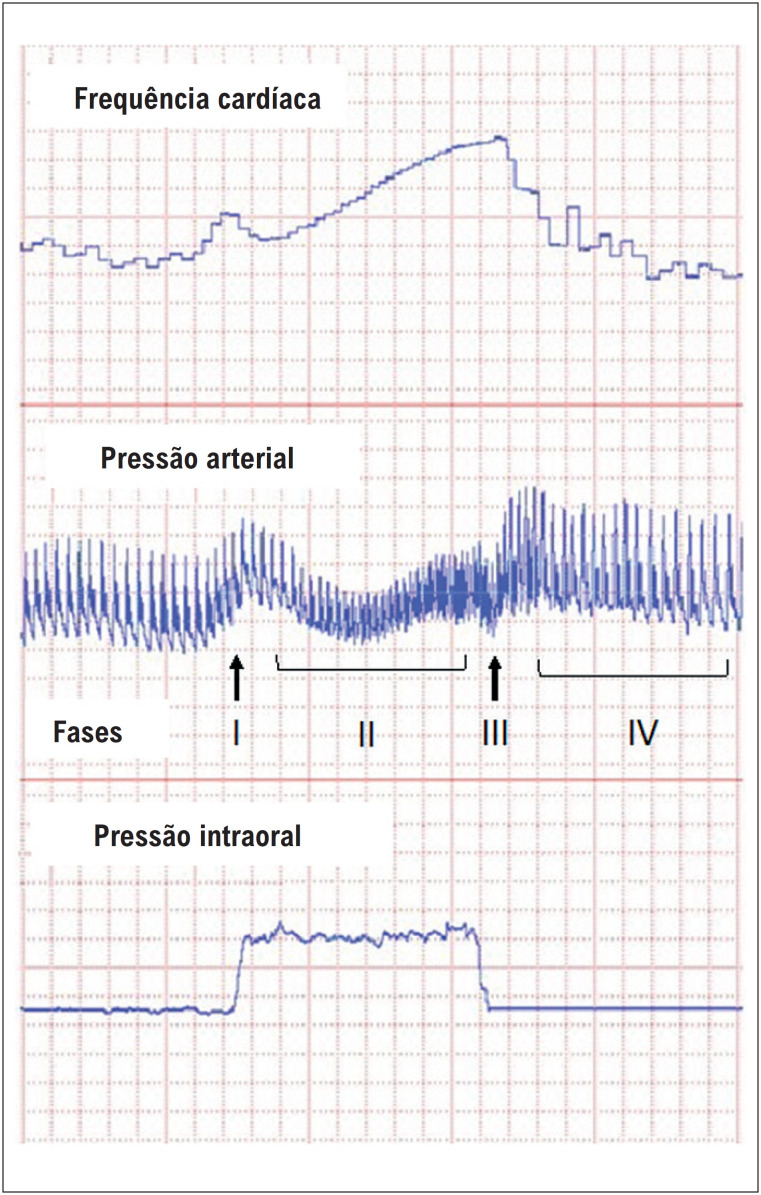
Registro gráfico da frequência cardíaca, pressão arterial e pressão intraoral durante um procedimento típico de manobra de Valsalva. Ao longo da fase II, ocorre uma diminuição da pressão arterial e taquicardia, durante as quais as alterações hemodinâmicas podem provocar aumento ou revelar gradiente da VSVE em pacientes com miocardiopatia hipertrófica.

Durante a fase de esforço expiratório da manobra de Valsalva (fase II), ocorre aumento da pressão intratorácica com consequente diminuição do retorno venoso ao coração, causando redução da pré-carga e queda progressiva da pressão arterial com ativação simpática reflexa causando taquicardia e estimulação adrenérgica miocárdica. A consequente redução do volume diastólico do ventrículo esquerdo e o aumento da contratilidade miocárdica podem provocar um aumento dinâmico da obstrução da VSVE.

Embora, na prática diária, a manobra de Valsalva seja geralmente realizada por meio de uma expiração forçada contra o dorso da mão do paciente por 10 a 20 segundos,^6^ a execução correta e adequada da manobra de Valsalva, e consequentemente seus efeitos hemodinâmicos, exige cuidado e padronização.

Inicialmente, solicita-se ao paciente que realize uma inspiração completa, seguida de um esforço expiratório contra uma resistência representada por um dispositivo bucal conectado a um manômetro aneroide (fase I, Figura 2). Uma manobra de Valsalva controlada significa que, durante a fase de esforço (fase II), o paciente deve atingir e manter uma pressão de 40 mmHg com duração de 10 a 20 segundos, monitorada por um manômetro. No entanto, apesar de simples e de baixo custo, estes aparelhos não estão disponíveis na grande maioria dos laboratórios de ecocardiografia.^6^ A manobra de Valsalva controlada é mais eficaz na elevação dos gradientes da VSVE do que as manobras não controladas.^7^ Sugerimos a utilização de um sistema simples adaptado de fluxômetro respiratório conectado a um pequeno manômetro, que pode ser visualizado pelo paciente durante a realização do exame ecocardiográfico (Figura 2). Os pacientes podem ser rapidamente treinados para atingir a pressão e mantê-la durante o período de 10 a 20 segundos.

**Figura 2:**
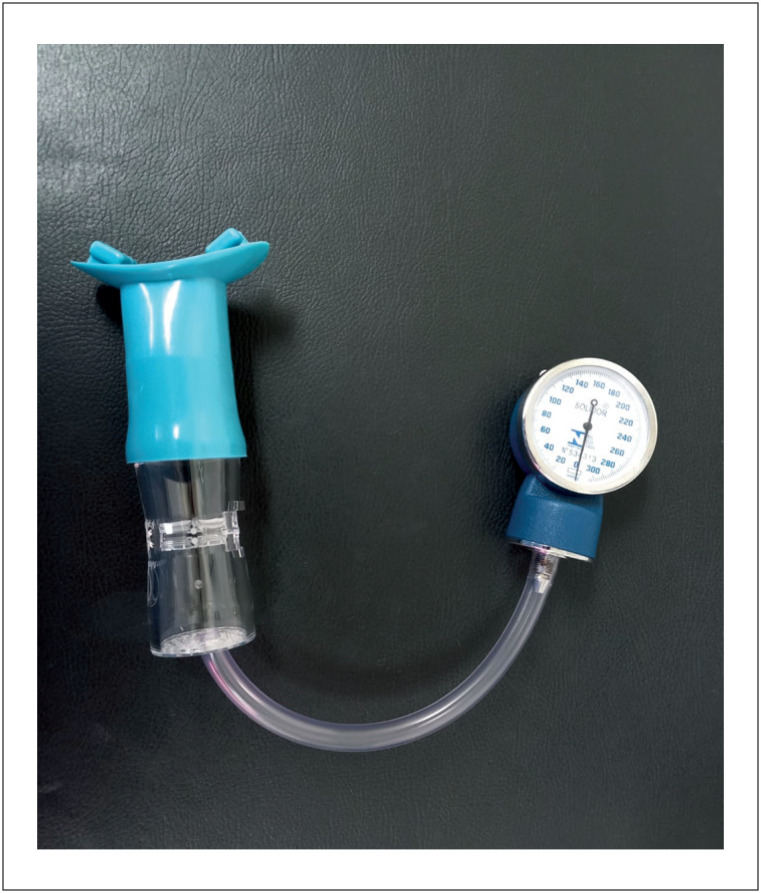
Aparelho simples para avaliação da pressão intraoral durante a manobra de Valsalva, composto por um manômetro conectado a um tubo bucal. Imagem cedida por Julio Cesar Crescencio da Faculdade de Medicina de Ribeirão Preto - Universidade de São Paulo.

Clinicamente, uma manobra de Valsalva adequada também pode ser avaliada pela distensão das veias do pescoço, rubor facial e aumento do tônus muscular abdominal. Durante a análise ecocardiográfica, a manobra de Valsalva adequada pode ser evidenciada pela redução do pico de velocidade da onda E do fluxo mitral de 20 cm/s na fase de esforço e pelo desvio do septo interatrial para a esquerda na fase de liberação.^5^

Quando não controlada, a manobra de Valsalva pode ser ineficaz e frequentemente observada em uma proporção significativa de pacientes. Isso pode ser explicado pela má compreensão da parte do paciente e por limitações clínicas, como comprometimento neurológico, pulmonar ou cognitivo. Além disso, a inspiração antes do esforço pode limitar a janela acústica e a análise ecocardiográfica.^5^

Outras manobras provocativas potencialmente úteis em pacientes com MCH incluem: 1) *squat-to-stand*: o paciente agacha por 3 segundos e depois fica em pé, repetindo o ciclo aproximadamente 5 vezes, o que também resulta em diminuição do retorno venoso; 2) inalação de nitrito de amila, que promove vasodilatação, diminui a pós-carga e aumenta a frequência cardíaca; e 3) ecocardiografia sob estresse físico com esteira ou bicicleta. O nitrito de amila não está disponível na grande maioria dos hospitais do Brasil. A ecocardiografia sob estresse físico é a manobra mais fisiológica e eficaz para avaliar a obstrução dinâmica da VSVE. Quando comparados, os testes provocativos em esteira e a manobra de Valsalva são capazes de provocar maior aumento nos gradientes da VSVE do que a manobra de *squat-to-stand*.^6^ Estudos anteriores demonstraram que uma proporção de 50% a 70% de indivíduos previamente medicados (com betabloqueadores ou bloqueadores dos canais de cálcio) atinge gradientes provocados superiores a 50 mmHg.^7^ No entanto, a ecocardiografia sob estresse físico ainda não está amplamente disponível.

Por outro lado, apesar de sua disponibilidade, a ecocardiografia sob estresse com dobutamina (EED) não é recomendada em pacientes com MCH. A EED pode induzir gradiente da VSVE, mas não é uma abordagem fisiológica e pode resultar em gradientes dinâmicos, mesmo em alguns indivíduos normais.^6^

Com base nos princípios descritos acima, todos os pacientes sintomáticos com MCH e sem gradientes obstrutivos significativos da VSVE em repouso devem ser testados com exames ecocardiográficos utilizando manobras provocativas. Sugerimos que todos sejam testados com manobra de Valsalva controlada (Figura 2) e, caso os gradientes não atinjam 50 mmHg, o teste seja continuado com modalidades de estresse sob esforço físico. Os relatórios ecocardiográficos devem incluir os gradientes da VSVE em repouso, comparados com os gradientes da VSVE provocados e uma descrição completa de qual teste foi realizado.

Concluindo, a manobra de Valsalva é uma ferramenta importante na ecocardiografia, especialmente na avaliação de pacientes com MCH para revelar a presença de obstrução dinâmica da VSVE. Deve ser realizada em todos os pacientes com formas MCH, nas formas obstrutiva ou não obstrutiva, como parte da avaliação inicial, principalmente em pacientes sem gradiente grave da VSVE em repouso. Porém, há grande variabilidade na maneira como a manobra de Valsalva é realizada, resultando em efeitos hemodinâmicos variáveis. Idealmente, a eficácia da manobra de Valsalva deve ser monitorizada e avaliada através de um manômetro em todos os pacientes; entretanto, na ausência de um aparelho adequado, a manobra de Valsalva deve ser executada com o maior rigor possível para atingir pressão e duração adequadas.^8^

## References

[1] Valsalva AM (1704). De Aure Humana Tractatus.

[2] Derbes VJ, Kerr A (1955). Valsalva’s Maneuver and Weber’s Experiment. N Engl J Med.

[3] Weber EF (1853). Sur Des Essais D’arrêt Volontaire de la Circulation… Et Du Coeur. Arch Ge´n de Me´d.

[4] Junqueira LF (2008). Teaching Cardiac Autonomic Function Dynamics Employing the Valsalva (Valsalva-Weber) Maneuver. Adv Physiol Educ.

[5] Ghazal SN (2017). Valsalva Maneuver in Echocardiography. J Echocardiogr.

[6] Nagueh SF, Phelan D, Abraham T, Armour A, Desai MY, Dragulescu A (2022). Recommendations for Multimodality Cardiovascular Imaging of Patients with Hypertrophic Cardiomyopathy: An Update from the American Society of Echocardiography, in Collaboration with the American Society of Nuclear Cardiology, the Society for Cardiovascular Magnetic Resonance, and the Society of Cardiovascular Computed Tomography. J Am Soc Echocardiogr.

[7] Kumar S, van Ness G, Bender A, Yadava M, Minnier J, Ravi S (2018). Standardized Goal-Directed Valsalva Maneuver for Assessment of Inducible Left Ventricular Outflow Tract Obstruction in Hypertrophic Cardiomyopathy. J Am Soc Echocardiogr.

[8] Joshi S, Patel UK, Yao SS, Castenada V, Isambert A, Winson G (2011). Standing and Exercise Doppler Echocardiography in Obstructive Hypertrophic Cardiomyopathy: The Range of Gradients with Upright Activity. J Am Soc Echocardiogr.

