# Supplementary material for: A Importância da Manobra de Valsalva Eficaz durante a Ecocardiografia na Miocardiopatia Hipertrófica
Source: Arq Bras Cardiol. 2024 Jul 1;121(7):e20230871. [Article in Portuguese] doi: 10.36660/abc.20230871 (PMC12080602; doi:10.36660/abc.20230871)
Supplement: Translation

# Translation: The Importance of the Effective Valsalva Maneuver during Echocardiography in Hypertrophic Cardiomyopathy

**DOI:** 10.36660/abc.20230871i

**Authors:** Viviane Tiemi Hotta, Minna Moreira Dias Romano, Silvio Henrique Barberato, Marcelo Luiz Campos Vieira, Fábio Fernandes, Marcus Vinicius Simões

**Affiliations:** 1 São Paulo SP Brazil Instituto do Coração do Hospital das Clínicas da Faculdade de Medicina da Universidade de São Paulo, São Paulo, SP – Brazil; 2 Ribeirão Preto SP Brazil Universidade de São Paulo Faculdade de Medicina de Ribeirão Preto, Ribeirão Preto, SP – Brazil; 3 Curitiba PR Brazil CardioEco Centro de Diagnóstico Cardiovascular, Curitiba, PR – Brazil; 4 Curitiba PR Brazil Quanta Diagnóstico – Ecocardiografia, Curitiba, PR – Brazil; 5 São Paulo SP Brazil Hospital Israelita Albert Einstein – Ecocardiografia Adultos, São Paulo, SP – Brazil

**Keywords:** Hypertrophic Cardiomyopathy, Echocardiography, Valsalva Maneuver

The Valsalva maneuver (VM) was first described in 1704 as a forceful expiration against the closed nose and glottis.^1,2^ At that time, no observations of the circulatory effects were mentioned. In 1853, Weber et al. described the hemodynamic and circulatory effects related to the VM, and it has been widely used in cardiology since then.^3^ The initial applications of the VM focused on the investigation of cardiac autonomic reflex control.^4^

Currently, the VM is recommended in echocardiography, in specific scenarios, such as in patients with hypertrophic cardiomyopathy (HCM), for evaluation of dynamic left ventricular outflow tract (LVOT) obstruction. Nevertheless, its execution varies greatly, and there is a lack of standardization of pressure control and time of execution.^5^

Particularly, in patients with HCM, it is crucial to evaluate the LVOT gradient using provocative tests in patients without significant LVOT obstruction at rest, since the large majority of symptomatic patients with HCM exhibits resting or provoked LVOT obstruction as a fundamental mechanism of dyspnea, which may be a relevant therapeutic target.

As LVOT obstruction is extremely influenced by left ventricular load conditions,^6^ the hemodynamic changes associated with the VM favor its use as a provocative test in patients with HCM. Figure 1 depicts the arterial blood pressure and heart rate changes during the different phases of the VM.

**Figure 1:** A graphical recording of the heart rate, arterial pressure, and intraoral pressure during a typical Valsalva maneuver procedure. Throughout phase II, there is a decrease in the arterial pressure and tachycardia, during which the hemodynamic changes may provoke an increase or unveil LVOT gradient in patients with hypertrophic cardiomyopathy.

During the expiratory strain phase of VM (phase II), there is an increase in intrathoracic pressure with a consequent decrease of venous return to the heart, causing preload reduction and progressive arterial pressure drop with reflex sympathetic activation causing tachycardia and adrenergic myocardial stimulation. The consequent reduction in left ventricular diastolic volume and increased myocardial contractility may provoke a dynamic increase in LVOT obstruction.

Even though, in daily practice, the VM is usually performed by a forceful expiration against the back of the patient’s hand for 10 to 20 seconds,^6^ the correct and adequate performance of VM, and consequently its hemodynamic effects, demands careful execution and standardization.

Initially, the patient is asked to take a full inspiration, followed by an expiratory effort against a resistance represented by a mouthpiece tube connected to an aneroid manometer (phase I, Figure 2). A controlled VM means that, during the strain phase of VM (phase II), the patient should achieve and maintain a pressure of 40 mmHg lasting 10 to 20 seconds, monitored by a pressure gauge. Nevertheless, although they are simple and inexpensive, these devices are not available in the great majority of echocardiography laboratories.^6^ Controlled VM is more effective in raising LVOT gradients than uncontrolled maneuvers.^7^ We suggest the use of an adapted simple system of respiratory flowmeter connected to a little manometer, which can be seen by the patient while performing echocardiography examination (Figure 2). Patients can be rapidly trained to achieve the pressure and to maintain it for 10 to 20 seconds.

**Figure 2:** Simple device for intraoral pressure assessment during the Valsalva maneuver, consisting of a pressure gauge connected to a mouthpiece tube. Courtesy of Julio Cesar Crescencio of the Faculty of Medicine, Ribeirão Preto, Universidade de São Paulo.

Clinically, an adequate VM can be also assessed by distention of neck veins, flushing of the face, and increased abdominal muscle tone. During echocardiographic analysis, adequate VM can be evidenced by a reduction in mitral inflow peak E velocity of 20 cm/s in the strain phase and interatrial septum shift to the left in the release phase.^5^

When it is not controlled, ineffective VM is often observed in a significant proportion of patients. This can be explained by poor patient comprehension and clinical limitations, such as neurologic, pulmonary, or cognitive impairment. Additionally, inspiration prior to strain can limit the imaging window and echocardiographic analysis.^5^

Other potentially useful provocative maneuvers in HCM patients include the following: 1) squat-to-stand: squat for 3 seconds and then stand repeated for approximately 5 cycles, which also results in decreased venous return; 2) amyl nitrite inhalation, which promotes vasodilation, decreases afterload, and increases heart rate; and 3) exercise stress echocardiography with a treadmill or a bicycle. Amyl nitrite it is not available in the great majority of hospitals in Brazil. Exercise stress echocardiography is the most physiological and effective maneuver to evaluate dynamic LVOT obstruction. When compared, provocative treadmill tests and VM are able to provoke a greater increase in LVOT gradients than squat-to-stand maneuver.^6^ Previous studies have shown that a proportion of 50% to 70% of previously medicated individuals (with beta-blockers or calcium channel blockers) achieve provoked gradients greater than 50 mmHg.^7^ However, exercise stress echocardiography is not widely available yet.

On the other hand, despite its availability, dobutamine stress echocardiography (DSE) is not recommended in patients with HCM. DSE can induce LVOT gradient; however, it is not a physiological approach, and it can result in dynamic gradients, even in some normal subjects.^6^

Based on the principles described above, all symptomatic patients with HCM and no significant resting LVOT obstructive gradients should be tested with echocardiographic examinations using provocative maneuvers. We suggest that all of them should be tested with a controlled VM (Figure 2), and, if gradients do not achieve 50 mmHg, testing should be continued with exercise stress. Echocardiographic reports should include resting LVOT gradients, compared to provoked LVOT gradients and a complete description of what test was performed.

In conclusion, the VM is an important tool in echocardiography, especially in the evaluation of patients with HCM to unveil the presence of dynamic LVOT obstruction. It must be performed in all patients with obstructive or non-obstructive forms of HCM as part of the initial evaluation, mainly in patients without severe LVOT gradient at rest. However, there is great variability in how the VM is performed, resulting in variable hemodynamic effects. Ideally, the efficacy of the VM should be monitored and assessed by using a pressure gauge in all patients; however, in the absence of an adequate device, the VM should be executed as rigorously as possible to achieve adequate pressure and duration.^8^
